# Study of the association of work-related musculoskeletal disorders and anxiety-depressive diseases

**DOI:** 10.1192/j.eurpsy.2023.2044

**Published:** 2023-07-19

**Authors:** L. Ben Afia, D. Brahim, I. Youssef, S. Ernez, W. Ayed, M. Mersni, N. Mechergui, N. Ladhari

**Affiliations:** Occupational pathology and fitness for work, Charles Nicolle Hospital, Tunis, Tunisia

## Abstract

**Introduction:**

Mental disorders, musculoskeletal diseases (MSDs) and their comorbidities are major threats to work and functional ability. The relationship between mental health and the common MSDs has not received enough attention

**Objectives:**

To study the socio-professional characteristics of workers suffering from work related MSD

To evaluate the association of work related MSDs with anxiety and depression disorders

**Methods:**

A descriptive cross-sectional study conducted among workers with work-related MSDs who consulted the occupational medicine department of the Charles Nicolle Hospital between January 2022 and September 2022. A remote survey was conducted among these workers to screen for anxiety and depressive disorders using the Hospital anxiety and Depressive Scale

**Results:**

The study population consisted of 54 workers with MSDs with a sex ratio (M/F) of 0.74. The average age was 44.4 [27-61 years]. The average professional seniority was 14.9 years±7 years and the sectors with the highest prevalence of MSDs were the health sector (22%), the food industry (13%) and the textile industry (11%). The workers reported MSDs of the lumbar spine in 61%, gonarthrosis in 31%, followed by MSDs of the upper limb in 25%. The prevalence of anxiety and depressive disorders were respectively 46% and 38%. There was no significant association between socio-demographic factors and anxiety depressive disorders. The anxiety disorder was associated with MSDs of the lumbar spine (p: 0.05; OR: 0.32 CI95% [0.1-1.09]).

**Conclusions:**

Anxiety and depressive disorders were common among workers with MSDs related to work. Interventions targeting psychological distress and work-related psychosocial characteristics may reduce their musculoskeletal pain.

**Disclosure of Interest:**

None Declared

